# Supplementary material for: Influence of different data-averaging methods on mean values of selected variables derived from preoperative cardiopulmonary exercise testing in patients scheduled for colorectal surgery
Source: PLoS One. 2023 Mar 16;18(3):e0283129. doi: 10.1371/journal.pone.0283129 (PMC10019694; doi:10.1371/journal.pone.0283129)

Supporting information S1, Graphical display of the Wasserman plots of patient 21 with the different data-averaging intervals.

Data visualization using 10 seconds data-averaging

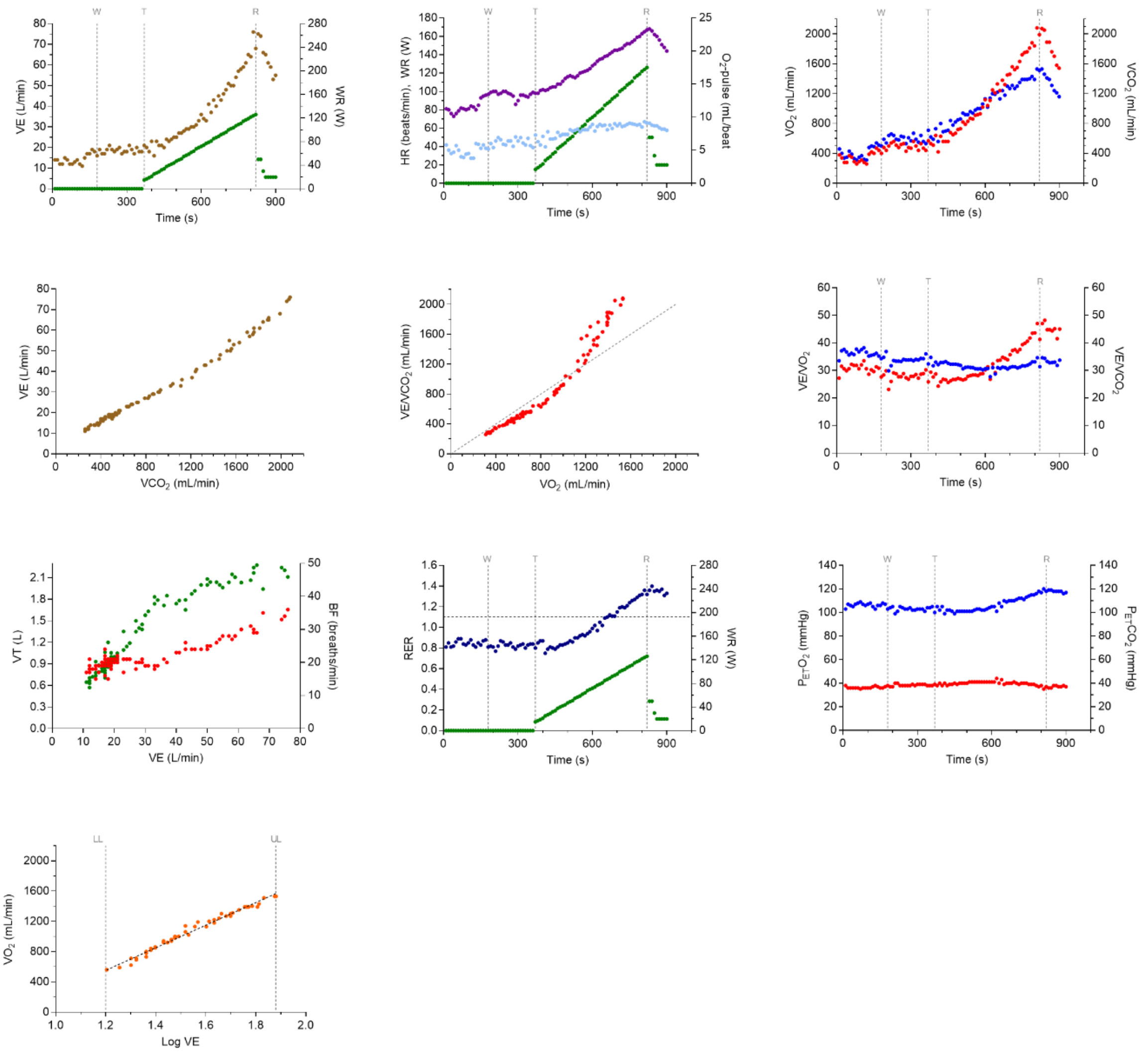

Data visualization using 20 seconds data-averaging

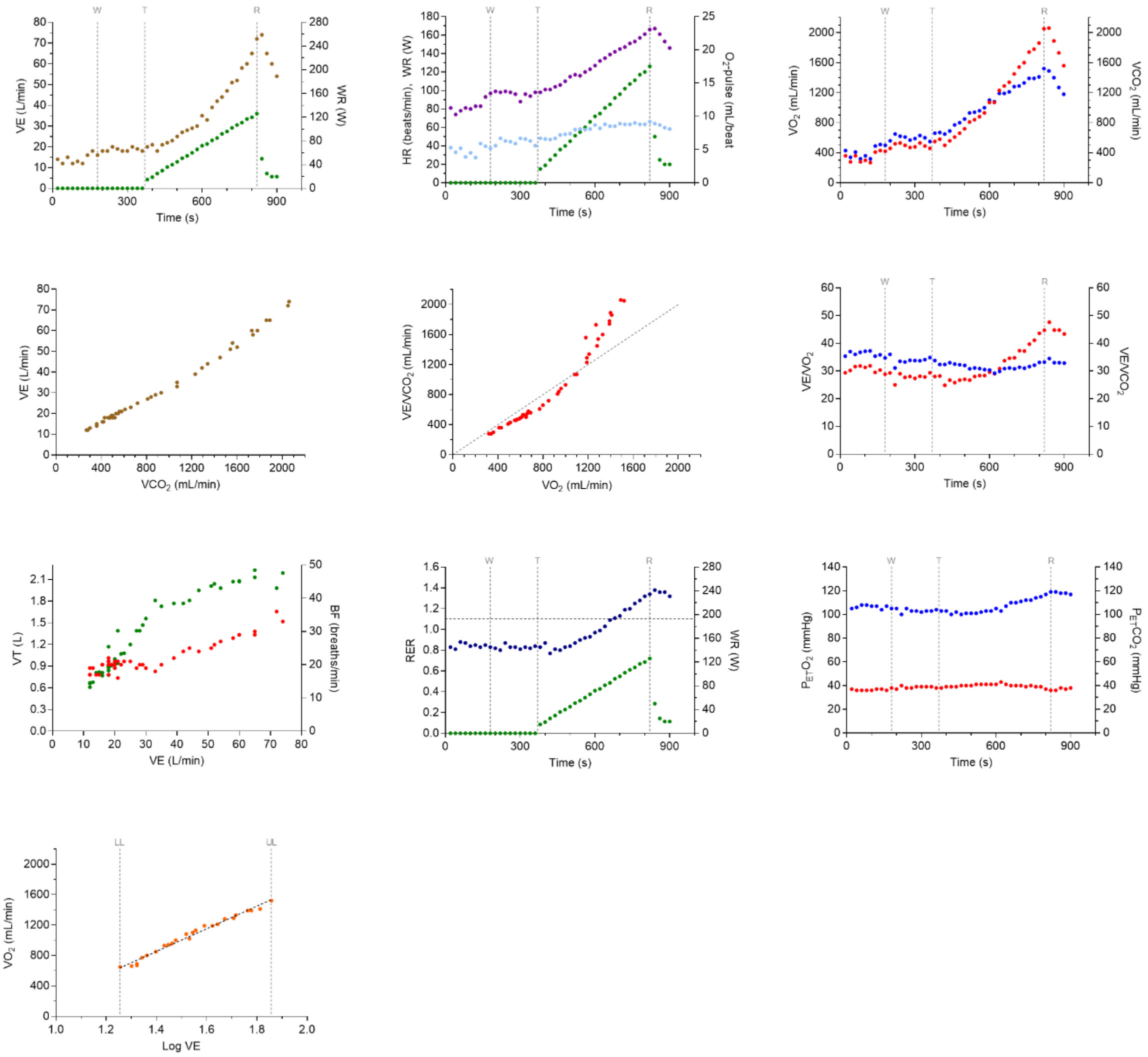

Data visualization using 30 seconds data-averaging

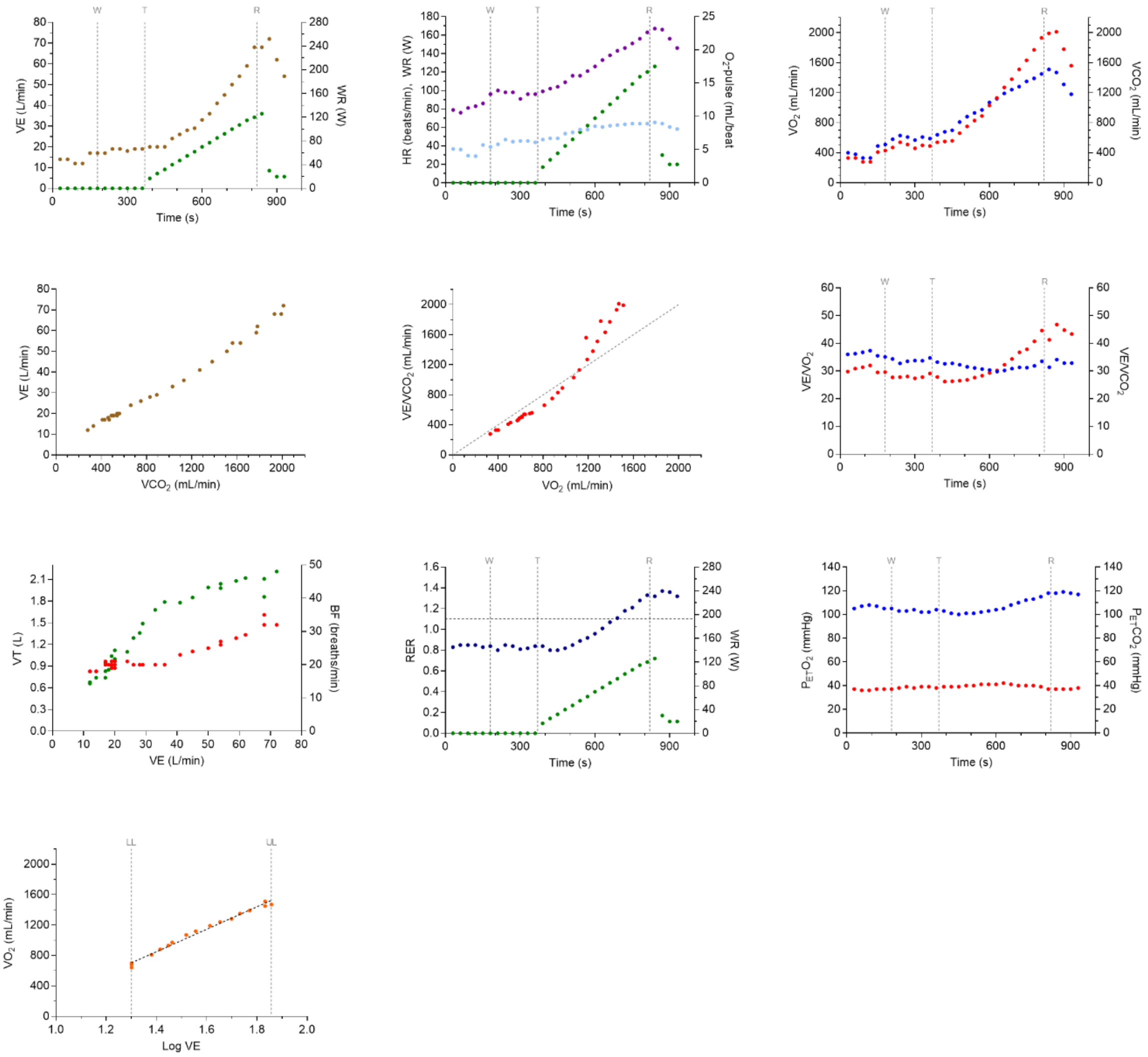

Data visualization using 3 breaths data-averaging

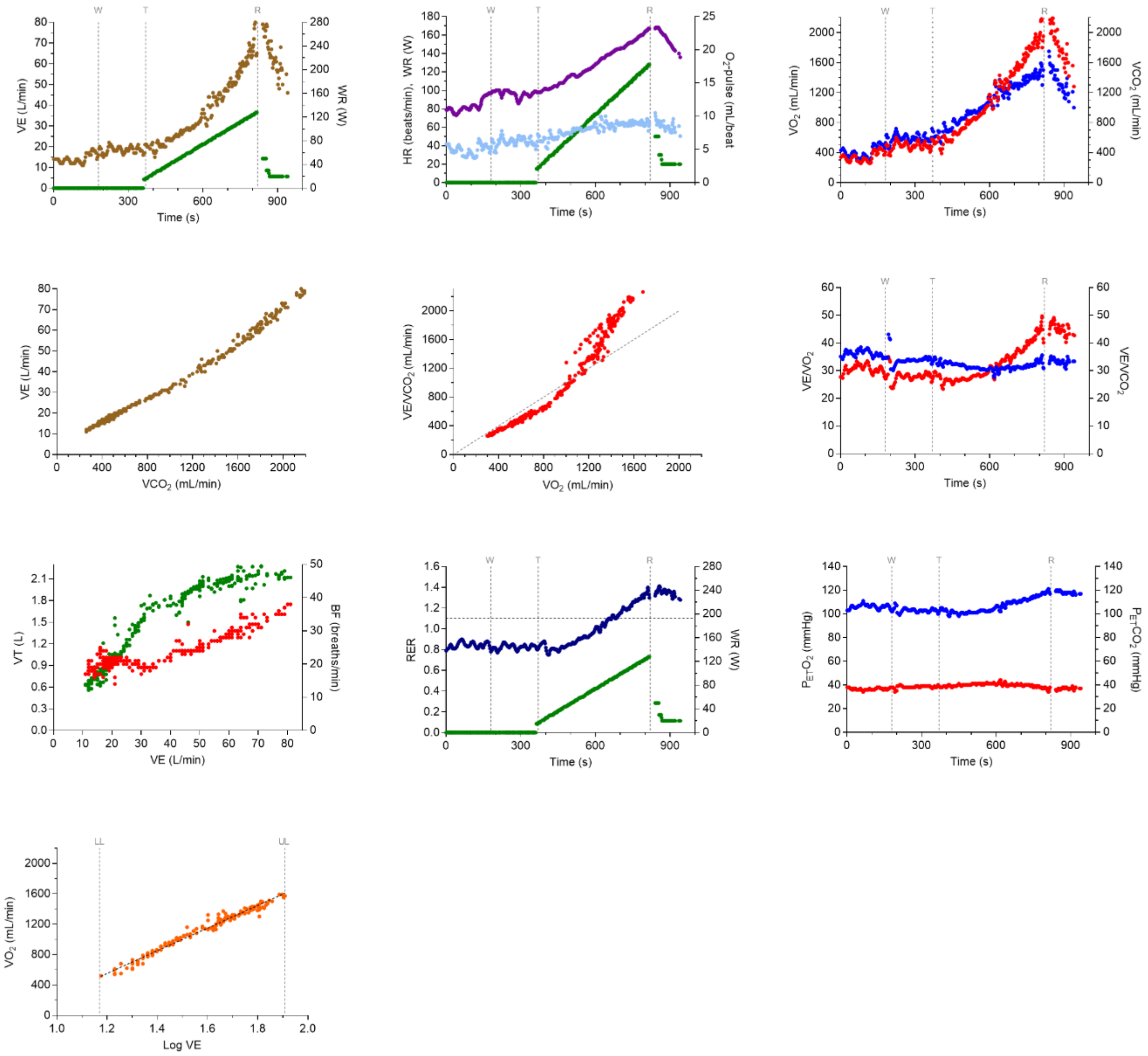

Data visualization using 7 breaths data-averaging

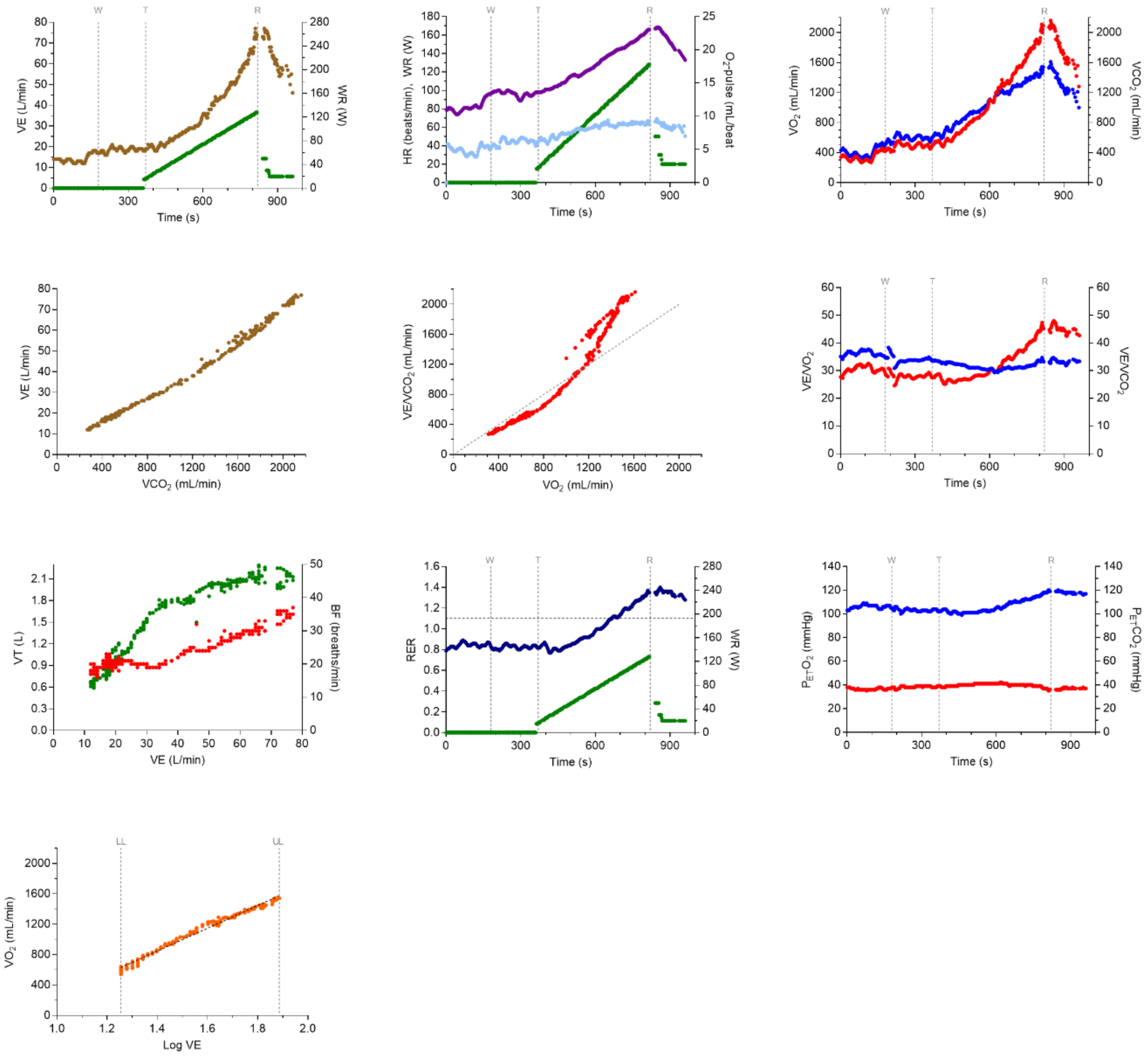

Supplement: S1 File — (PDF) [file pone.0283129.s001.pdf]
