# Supplementary material for: Influence of different data-averaging methods on mean values of selected variables derived from preoperative cardiopulmonary exercise testing in patients scheduled for colorectal surgery
Source: PLoS One. 2023 Mar 16;18(3):e0283129. doi: 10.1371/journal.pone.0283129 (PMC10019694; doi:10.1371/journal.pone.0283129)
Supplement: S2 File — (PDF) [file pone.0283129.s002.pdf]

Supporting information S2, Guideline for systematic interpretation of preoperative cardiopulmonary exercise testing.

Contents

1. Ventilatory anaerobic threshold

2. Respiratory compensation point

3. Oxygen uptake at peak exercise

4. Slope of the relationship between minute ventilation and carbon dioxide production

5. Oxygen uptake efficiency slope

6. References

1. Ventilatory anaerobic threshold

The determination of the ventilatory anaerobic threshold (VAT) – *synonyms: ventilatory threshold, gas exchange threshold, aerobic threshold, anaerobic threshold, and VT1* – is based on 3 criteria.

Criterion 1
Identify an increase in carbon dioxide production (VCO_2) relative to the oxygen uptake (VO_2) above the VAT by using the V-slope or modified V-slope method.

1. Go to the plot in which the VCO_2 (y-axis) and VO_2 (x-axis) are plotted against each other (see **Figure 1**).

2. Use:

   a. The *V-slope method:*
   Identify the intersection of the regression lines **S1** (below the VAT) and **S2** (above the VAT) in the VCO_2-VO_2 relationship.

   Or

   b. The *modified V-slope method:*
   Move a line with a gradient of 1.0 from the lower right-hand corner of the graph towards the VCO_2-VO_2 relationship and identify the point at which this line first touches the curve of the relationship between VCO_2 and VO_2.

   (Note: take outliers into account)

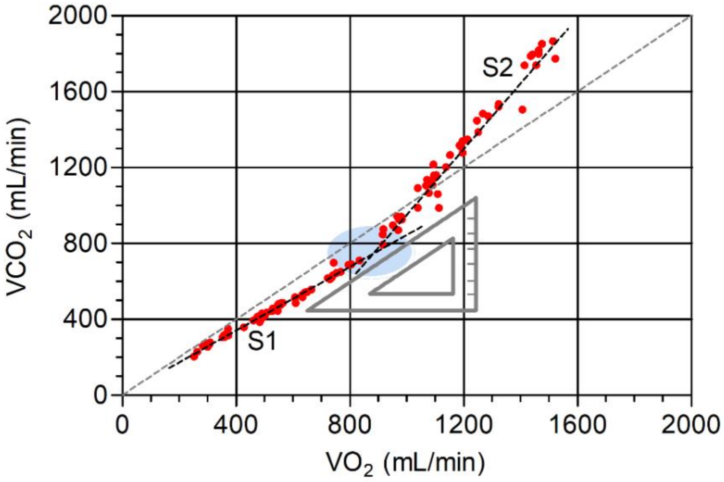

Figure 1. VCO_2 against VO_2.
S1 = slope below the VAT; S2 = slope above the VAT.

Criterion 2
Identify hyperventilation relative to the VO_2 using the ventilatory equivalents method.

1. Go to the plot in which the ventilatory equivalent for oxygen (VE/VO_2) and ventilatory equivalent for carbon dioxide (VE/VCO_2) (both on the y-axis) are plotted against time (x-axis) (see **Figure 2**).

2. Identify the point at which the VE/VO_2 ratio begins to rise after an initially flat or decreasing period and does not return to baseline.

3. For verification, go to the plot with the partial end-tidal oxygen tension ($P_{ET}O_2$) and partial end-tidal carbon dioxide tension ($P_{ET}CO_2$) (both on the y-axis) are plotted against time (x-axis) (see **Figure 3**): check whether $P_{ET}O_2$ starts to rise at this point after an initially flat or decreasing period and does not return to baseline.

   (Note: take outliers into account)

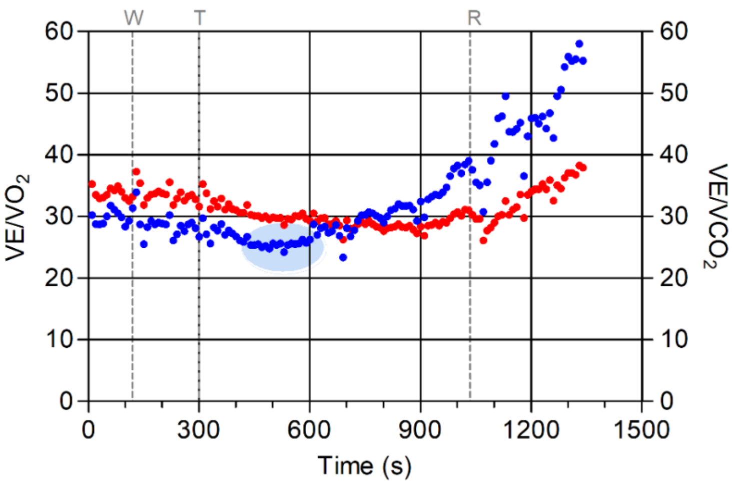

Figure 2. VE/VO_2 ● and VE/VCO_2 ● against time.
W = start warm-up; T = start test; R = start recovery.

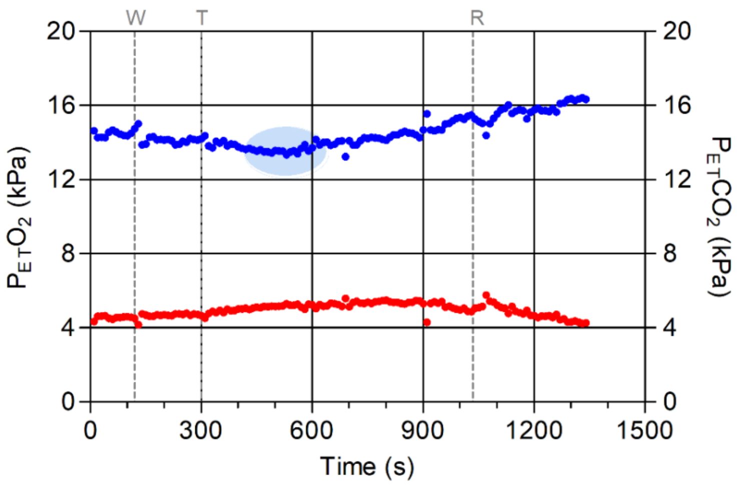

Figure 3. $P_{ET}O_2$ ● and $P_{ET}CO_2$ ● against time.
W = start warm-up; T = start test; R = start recovery.

Criterion 3

Check whether the VAT identified using criteria 1 and 2 is not caused by hyperventilation relative to VCO_2 using the ventilatory equivalents method.

1. Go to the plot in which VE/VO_2 and VE/VCO_2 (both on the y-axis) are plotted against time (x-axis) (see **Figure 2**).

2. Confirm that the VE/VCO_2 ratio remains constant or continues to decrease at the point at which VE/VO_2 starts to rise systematically.

3. For verification, go to the plot in which the $P_{ET}O_2$ and $P_{ET}CO_2$ (both on the y-axis) are plotted against time (x-axis) (see **Figure 3**): check if there is no reciprocal decrease in $P_{ET}CO_2$ at the point where $PETO_2$ starts to rise systematically.

   (Note: take outliers into account)

Use the 3 criteria above to identify the point that best represents the VAT.
(If you cannot identify a VAT based on these criteria, please select the option "VAT not determinable" in the drop-down menu of the Microsoft Excel document.)

2. Respiratory compensation point

The determination of the respiratory compensation point (RCP) – *synonyms: anaerobic threshold and VT2* – is based on 2 criteria.

Criterion 1
Identify the point at which the minute ventilation (VE) starts to increase more steep (respiratory compensation) in relation to the carbon dioxide production (VCO_2) due to metabolic acidosis.

1. Go to the plot in which VE (y-axis) and VCO_2 (x-axis) are plotted against each other (see **Figure 4**).

2. Identify the point where the slope of the relationship between VE and VCO_2 steepens.

   (Note: take outliers into account)

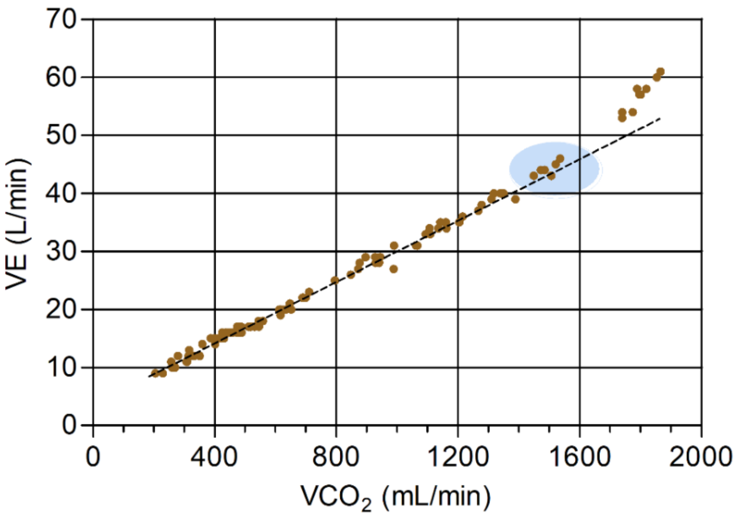

Figure 4. VE against VCO_2.

Criterion 2

Identify hyperventilation relative to the carbon dioxide production (VCO_2) using the ventilatory equivalents method.

1. Go to the plot in which the ventilatory equivalents for oxygen (VE/VO_2) and carbon dioxide production (VE/VCO_2) (both on the y-axis) are plotted against time (x-axis) (see **Figure 5**).

2. Identify the point at which the VE/VCO_2 ratio begins to rise after an initially flat or decreasing period and does not return to baseline.

3. For verification, go to the plot in which the partial end-tidal oxygen tension ($P_{ET}O_2$) and partial end-tidal carbon dioxide tension ($P_{ET}CO_2$) (both on the y-axis) are plotted against time (x-axis) (see **Figure 6**): at this point, $P_{ET}CO_2$ should begin to decline after an initially flat or increasing period.

(Note: take outliers into account)

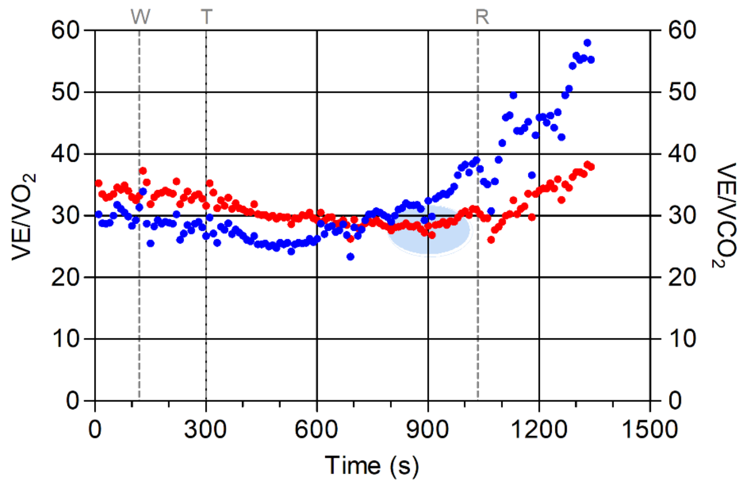

Figure 5. VE/VO_2 ● and VE/VCO_2 ● against time.
W = start warm-up; T = start test; R = start recovery.

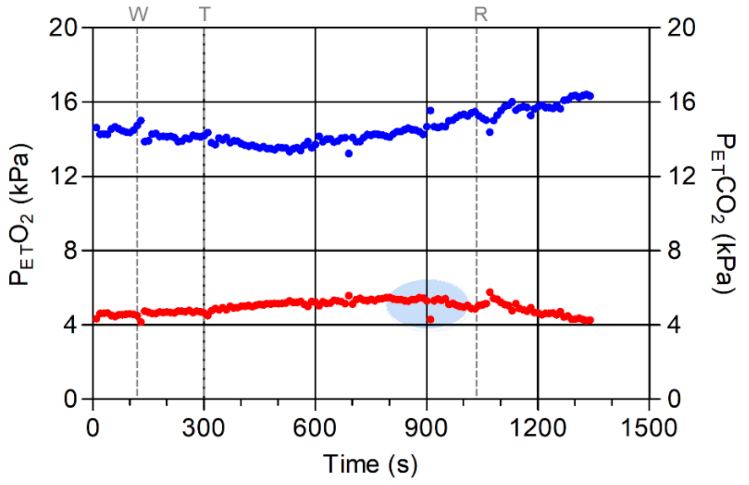

Figure 6. $P_{ET}O_2$ ● and $P_{ET}CO_2$ ● against time.
W = start warm-up; T = start test; R = start recovery.

Use the 2 criteria above to identify the point that best represents the RCP.
(If you cannot identify an RCP based on these criteria, please select the option "RCP not determinable" in the drop-down menu of the Microsoft Excel document.)

3. Oxygen uptake at peak exercise

Determine the oxygen uptake (VO_2) at peak exercise (VO_{2peak}) using the following steps.

Criterion
Determine whether a *valid* VO_{2peak} was achieved.

1. Verify whether the VO_{2peak} was attained during a (near) maximal effort by checking if:

   a. The achieved respiratory exchange ratio at peak exercise (RER_{peak}) was ≥1.10

   And/or

   b. The achieved heart rate at peak exercise (HR_{peak}) was >95% of predicted (*predicted HR_{peak} = 208 – (0.8 × age in years)*)

2. When the abovementioned criteria for a maximal effort are met, go to the plot in which the VO_2 and VCO_2 (both on the y-axis) are plotted against time (x-axis) (see **Figure 7**) to determine the VO_{2peak} as the average VO_2 value over the last 30 seconds of the test.

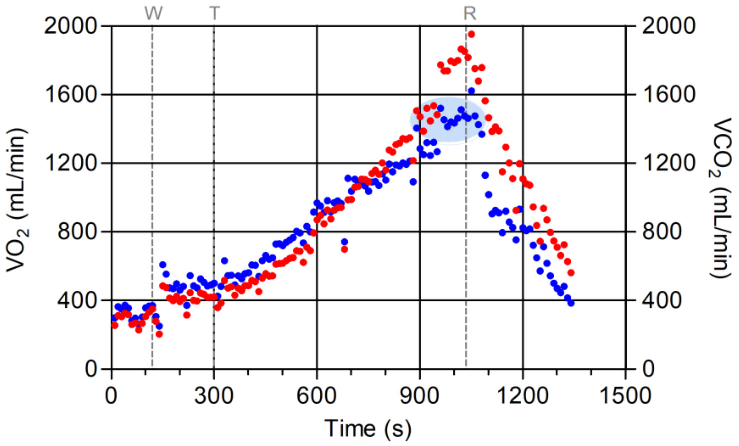

Figure 7. VO_2 ● and VCO_2 ● against time.
W = start warm-up; T = start test; R = start recovery.

Determine the VO_{2peak} (mL/min) averaged over the last 30 seconds of the test.
(If the abovementioned criteria for a maximal effort are not met, select the option “no valid VO_{2peak}” in the drop-down menu of the Microsoft Excel document.)

4. Slope of the relationship between minute ventilation and carbon dioxide production

Determine the relationship between minute ventilation (VE) and carbon dioxide production (VCO_2), called VE/VCO_2-slope, up to the respiratory compensation point (RCP) using the following steps:

1. Go to the plot in which the VE (y-axis) is plotted against VCO_2 (x-axis) (see **Figure 8**).

2. Place the “lower limit” (**LL**) line of the VE/VCO_2-relationship at the point at which the work rate starts to increase.

3. Place the “upper limit” (**UL**) at the point at which the slope of the relationship between the VE and VCO_2 steepens (loss of linearity): if no RCP can be determined, the UL line should be placed at the end of the exercise phase (peak exercise).

   (Note: take outliers into account)

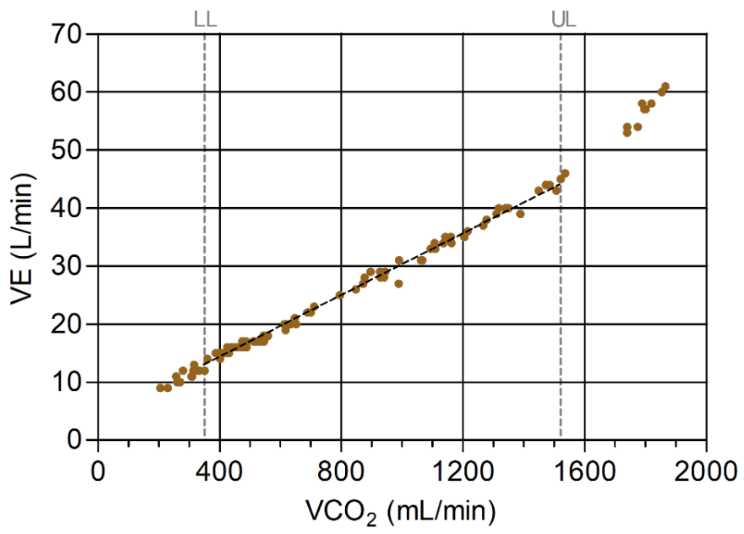

Figure 8. VE against VCO_2.
LL = lower limit; UL = upper limit.

The slope of the regression line describing the relationship of the VE with VCO_2 between the LL and UL represents the VE/VCO_2-slope.

5. Oxygen uptake efficiency slope

Determine the oxygen uptake efficiency slope (OUES) from the point at which the work rate starts to increase up to a plateau in oxygen uptake (VO_2) despite an increase in work rate (a 'true' VO_{2max}) or, in case no VO_2-plateau can be observed, up to the end of the exercise phase (peak exercise) using the following steps.

1. Go to the plot in which the VO_2 (y-axis) is plotted against the logarithm of the minute ventilation (Log VE) (x-axis) (see **Figure 9**).

2. Place the "lower limit" (**LL**) line at the first data point in the graph (left side), the start of the exercise phase.

3. Place the "upper limit" (**UL**) line at the start of the VO_2-plateau, or, in case there is no VO_2-plateau, at the end of the exercise phase (peak exercise).

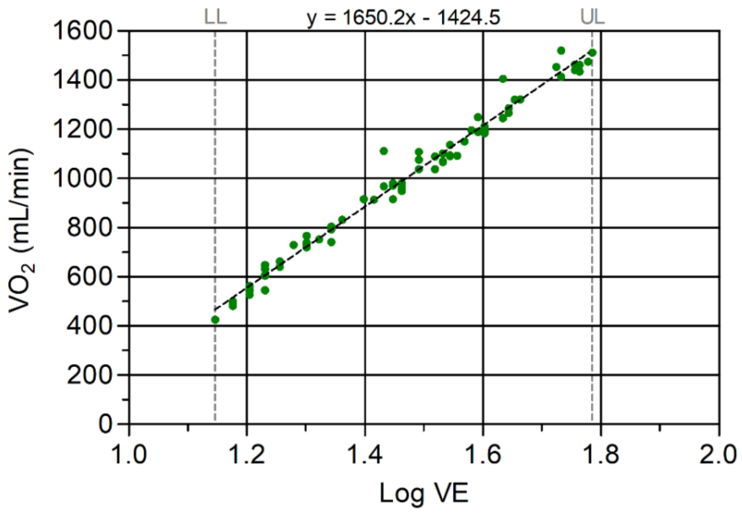

Figure 9. VO_2 against Log VE.
LL = lower limit; UL = upper limit.

The regression coefficient of the regression line between the LL en UL represents the OUES.

The OUES is determined with the formula: VO_2 = (a × Log VE) + b.
The regression coefficient "a" (1650.2 in the example in Figure 8) represents the increase in oxygen uptake (VO_2) relative to the logarithm of the minute ventilation (VE) and is called the OUES.

6. References

- American Thoracic Society; American College of Chest Physicians. ATS/ACCP statement on cardiopulmonary exercise testing. Am J Respir Crit Care Med. 2003;167:211-77.

- Bjørke ACH, Raastad T, Berntsen S. Criteria for the determination of maximal oxygen uptake in patients newly diagnosed with cancer: baseline data from the randomized controlled trial of physical training and cancer (Phys-Can). PLoS One. 2020;15:e0234507.

- Bongers BC, Berkel AE, Klaase JM, van Meeteren NL. An evaluation of the validity of the pre-operative oxygen uptake efficiency slope as an indicator of cardiorespiratory fitness in elderly patients scheduled for major colorectal surgery. Anaesthesia. 2017;72:1206-16.

- Dumitrescu D, Rosenkranz S. Graphical Data display for clinical cardiopulmonary exercise testing. Ann Am Thorac Soc. 2017;14(Supplement_1):S12-S21.

- Levett DZH, Jack S, Swart M, Carlisle J, Wilson J, Snowden C, Riley M, Danjoux G, Ward SA, Older P, Grocott MPW; Perioperative Exercise Testing and Training Society (POETTS). Perioperative cardiopulmonary exercise testing (CPET): consensus clinical guidelines on indications, organization, conduct, and physiological interpretation. Br J Anaesth. 2018;120:484-500.

- Mezzani A, Agostoni P, Cohen-Solal A, Corrà U, Jegier A, Kouidi E, Mazic S, Meurin P, Piepoli M, Simon A, Laethem CV, Vanhees L. Standards for the use of cardiopulmonary exercise testing for the functional evaluation of cardiac patients: a report from the Exercise Physiology Section of the European Association for Cardiovascular Prevention and Rehabilitation. Eur J Cardiovasc Prev Rehabil. 2009;16:249-67.
