# Supplementary material for: Influence of different data-averaging methods on mean values of selected variables derived from preoperative cardiopulmonary exercise testing in patients scheduled for colorectal surgery
Source: PLoS One. 2023 Mar 16;18(3):e0283129. doi: 10.1371/journal.pone.0283129 (PMC10019694; doi:10.1371/journal.pone.0283129)
Supplement: Attachment — Submitted filename: Response to the reviewers.docx [file pone.0283129.s004.docx]

Response to reviewers’ comments:

Reviewer #1: Interesting substudy of an approved study. appropriately conducted analysis

A few points to address :
The authors write as if assuming a degree of precision to interpretation, and hard differentation into low or high risk categories which isn`t typical of real life practice .
The authors have referenced the Rose “Grey Zone” paper
Rose GA, Davies RG, Davison GW, Adams RA, Williams IM, Lewis MH, et al. The 434 cardiopulmonary exercise test grey zone; optimising fitness stratification by application of critical 435 difference. Br J Anaesth. 2018;120(6):1187-94
…which makes this point well . The authors of the current study similarly make it clear in their conclusion that any apparent differences in values due to averaging are not clinically relevant. Important that they convey this clearly in the abstract

Authors’ response: We fully agree with the reviewer that preoperative risk assessment should be seen in the broader context of CPET interpretation and patient assessment in general.

We added **“or clinically relevant”** to the conclusion of the abstract

To avoid any unclarities regarding the point put forward by the reviewer about the conclusion in the abstract, we also adjusted the abstract as follows: The section **~~“the proportion of patients classified as high or low risk for complications.”~~** was deleted. And stated more in general as **“perioperative risk assessment.”**

Patients are generally not classified in a binary fashion as either high or low risk based on whether they are above or below strictly defined single threshold limits / Overall summary of risk tends to be a composite assessment taking into account many CPET measurements , & comorbidities.
The CPET is not applied as a pass or fail test . CPET derived Individualised Risk estimates are used to add weight to shared decision making about whether to proceed to surgery , and to help determine perioperative care pathway ( eg High Dependency Care versus ward based care pathway ) . It is worth covering this point about clinical application in the discussion

Authors’ response: In the manuscript, this point was briefly mentioned in the discussion on Page 21, Line 334 - 335. Nevertheless, we agree that we should elaborate more on this. Therefore, we adjusted this part of the discussion on page 21 Line 335-338 as follows:

~~Although it is recognized that risk assessment is not solely based on the numerical value of any CPET-derived variable~~

**“It is recognized that preoperative risk assessment is not solely based on risk thresholds determined by CPET, but rather consists of a composite assessment, taking into account the full CPET in combination with other preoperative risk factors such as, but not limited to, malnutrition, comorbidities, and geriatric status.”**

Section by section
Title : “ Influence of different data-averaging methods on preoperative risk assessment using
cardiopulmonary exercise testing in patients scheduled for colorectal surgery”. I think this title is over stating the impact of the study findings. More appropriate to say “ Influence of different data-averaging methods on mean values of selected variables derived from cardiopulmonary exercise testing in patients scheduled for colorectal surgery”

Authors’ response: We adjusted the title according to the suggestion of the reviewer. The new title reads as follows: **“Influence of different data-averaging methods on mean values of selected variables derived from preoperative cardiopulmonary exercise testing in patients scheduled for colorectal surgery”**

Abstract could define exactly which CPET derived variables were considered in the study – these are defined on p11 , para 2
Methods – clear
Results clear
Table 2 is really good to demonstrate the results
Figure 1 is a graphical representation of averaging – this is a powerful figure to illustrate the point that regardless of averaging method – the thresholds of interest are generally consistent
Figure 2 similarly is powerful to demonstrate the spread of individual patients` values dependent on the method of averaging

Authors’ response: We thank the reviewer for his positive evaluation of the manuscript and figures and the suggestion to define the exact variables in the abstract. We added the following to the methods section of the abstract: **“The variables of interest were oxygen uptake at peak exercise (VO_2peak_), oxygen uptake at the ventilatory anaerobic threshold (VO_2VAT_), oxygen uptake efficiency slope (OUES), the ventilatory equivalent for carbon dioxide at the ventilatory anaerobic threshold (VE/VCO_2VAT_), and the slope of the relationship between the minute ventilation and carbon dioxide production (VE/VCO_2_-slope).”**

P 11 para 2 – several CPET derived variables defined – however the abbrevations (VO2VAT) and(VE/VCO2VAT) are unusual . I`m accustomed to VO2AT and VE/VCO2AT.

Authors’ response: Indeed, many different terminologies are used within the literature to define CPET-derived thresholds. We prefer to use the term ventilatory anaerobic threshold (VAT) over anaerobic threshold (AT), as is it makes more clear that the thresholds are derived from respiratory gasses (as opposed to i.e., lactate measurement). Nevertheless, if the reviewer or the editor insist on using AT instead, we are willing to adjust the terminology throughout the manuscript.

Discussion :
Re-states main findings and discusses implications in context . Reasonable and moderate conclusions

Reviewer #2:

This study make nonsense. If the aim is to establish whether the averaging interval of VO2 data has an impact in the VO2max value and the medical decisions adopting depending on this value why are the authors analyzing all results using 30-s averages?

Authors’ response: We thank the reviewer for the critical appraisal of our study but we regret that the reviewer qualifies our study as nonsense. Before we can reflect on the reviewers points, we must emphasize that we did not analyze all results using 30-second data-averaging. In addition, we did not aim to establish whether data-averaging intervals impact VO_2peak_ values alone. Instead, we assessed many more variables (as outlined on page 10) using 5 different data averaging intervals. Therefore, the point the reviewer merely refers to the determination of VO_2peak_ values.

Regarding the determination of VO_2peak_ values, let us explain why we chose to analyze VO_2peak_ values over an interval close to 30-seconds regardless of the used data-averaging method. In most (if not all) software packages used for CPET interpretation, VO_2peak_ is calculated over a period that has to be manually set. That is, instead of using one point that is allocated as VO_2peak_, a lower limit and upper limit have to be set in order to demark the period over which VO_2peak_ is calculated (see also Figure 1, red shaded area in each graph). With this in mind, the method chosen in the current study mimics how VO_2peak_ is estimated in clinical practice. Nevertheless, we do agree with the reviewer that by doing so, the variability introduced by the data-averaging interval is attenuated as was already discussed on page 21, line 344-347. Nevertheless, this also reflects routine practice.

Moreover, the metabolic cart data are incomplete, please add PETO2 and PETCO2 data.

Authors’ response: We are not sure what the reviewer is referring to as we did not disclose any metabolic cart data. We did however include the raw data on which the analyses are based. Is that what the reviewer is referring to?

Are all metabolic carts used disclosed? Or all test were performed with the same metabolic cart?

Authors’ response: All tests are performed on the same metabolic cart as reported in the methods section in the paragraph “*Preoperative cardiopulmonary exercise testing”* on page 7 and 8. We included a data file with the raw data that was used for the analysis. We did not include the metabolic cart data as we believe that due to the relative small study sample it would be hard to guarantee anonymity of our participants. Nevertheless, on reasonable request the metabolic cart data could be shared after personal communication with the authors.

Please add as as an example the full output data of one of the incremental exercise test with breath by breath data and then the calculated 10, 20 or 30 s averaged values.

Authors’ response: We added an example of a graphical display of the metabolic cart of patient 21 as Supporting information S3.
