# Supplementary material for: Influence of different data-averaging methods on mean values of selected variables derived from preoperative cardiopulmonary exercise testing in patients scheduled for colorectal surgery
Source: PLoS One. 2023 Mar 16;18(3):e0283129. doi: 10.1371/journal.pone.0283129 (PMC10019694; doi:10.1371/journal.pone.0283129)
Supplement: Attachment — Submitted filename: Response to the reviewers.docx [file pone.0283129.s005.docx]

Response to reviewers’ comments:

**Reviewer #1:**

All comments have been addressed

Authors’ response:

We would like to thank the reviewer for his/her valuable contribution.

**Reviewer #3:**

The introduction is not specific to the findings of this study until line 108. Although you mention you have addressed reviewer #1 concerns about the interpretation of CPEX utility, comments like lines 106-107 are misleading. I absolutely agree that threshold values are far from agreed upon across surgical specialties. CPEX is at best a single test that should be interpreted in context. I do not believe that patients would be denied surgery on the result on this single test in many institutions - unless you have data on this. This should be made clearer in your paper, not just in the conclusion, which seems at odds now with the introduction.

Authors’ response:

We amended the introduction to make it more specific to the topic of the study. Therefore, we deleted the following section: “**In the Netherlands, approximately 12,000 men and women are diagnosed with colorectal cancer annually. The primary curative treatment for colorectal cancer is surgery. The incidence of postoperative complications is high, and are even as high as 60-70% in high-risk patients. Strategies aiming at risk reduction for postoperative complications and enhancing postoperative recovery by improving a patient’s health status in the preoperative period are called prehabilitation. Risk assessment is an essential part of the preoperative diagnostic work-up, as especially patients at high risk for postoperative complications might benefit from comprehensive prehabilitation to improve their postoperative outcomes. There is emerging evidence that prehabilitation can reduce the occurrence of postoperative complications up to approximately 50% in high-risk patients undergoing major abdominal surgery.”**

We agree with the reviewer that denying surgery solely based on CPET results is inappropriate and not in line with clinical practice. In our review CPET outcomes should mainly be used to optimize a patient’s health prior to surgery or guide postoperative care. To be clearer about our intentions we added the following to the introduction (**lines 63-65**) “…**inform collaborative decision-making, to optimize comorbidities, to triage perioperative care (e.g., ward, intensive care), to advice on preoperative physical exercise training (e.g., risk assessment, contraindications), and…”**

Also, the “misleading” section in (**lines 106 -107**) in the original manuscript) was adapted as follows (**lines 96-100):**

**“Although preoperative risk assessment should be multimodal, CPET-derived thresholds are often used to recognize patients with a low aerobic fitness who have a high risk for adverse surgical outcomes. In major abdominal surgery, often used thresholds to identify patients at high-risk for postoperative complications are a VO_2peak_ <18.2 mL/kg/min and/or a VO_2VAT_ <11.1 mL/kg/min.”**

I do believe that it is useful to the examine the influence of data-averaging methods for this test, which the authors have done well. This, in my opinion, should be the focus of the paper. I do not believe the secondary aim of this study is worthwhile. I understand the point being made, but clarifying the influence of different data averaging method is sufficient. We can interpret the clinical application ourselves. I therefore recommend changing the focus of the paper to reflect the above.

Authors’ response:

Before data analysis, we hypothesized and expected that the differences in the mean values of the CPET variables between the different data-averaging intervals would be larger. Therefore, we included the secondary aim to explore if any differences in the mean values of CPET-derived variables used for risk-assessment would translate in differences in the proportion of patients classified as high risk based on CPET results. We agree with the reviewer that the influence of data averaging intervals on the (mean) numerical values of CPET derived parameters is most informative. Therefore, this is the primary focus (and aim) of the paper. Given the results, we acknowledge the point made by the reviewer. Nevertheless, in our opinion, discarding the secondary aim based on the results of the study would not be appropriate. In addition, the secondary aim could be worthwhile for clinicians who are less familiar with preoperative risk assessment and / or CPET interpretation.

**Reviewer #4:**

General comments

The authors of this study addressed a relatively simple question, yet potentially important from a practical perspective, whether the way data from CPET is averaged affects parameters estimated from the CPET in a clinical population.

Overall, the manuscript is well written, and contains all technically relevant information.

I think the authors and previous reviewers have done a thorough job, and thus my comments remain relatively minor. I hope my comments help improve the manuscript.

The main comments (see below) refers to the interpretation of some of the results in the discussion.

Specific comments

Line 28 abstract – please abbreviate cardiorespiratory fitness as CRF.

Authors’ response:

The abbreviation “**CRF**” was added in the abstract (**line 28**).

Line 64-66 – perhaps you can make the point/link (or make this point clearer) that a key goal of prehabilitation is to increase CRF, and increases in CRF have a clinically relevant positive effect on subsequent complications.

Authors’ response:

We agree with the reviewer that improvement of cardiorespiratory fitness is generally believed to be one of the mechanisms by which prehabilitation might improve postoperative outcomes. However, the section the reviewer is referencing to was omitted due to comments of reviewer 3.

Line 100 and throughout the study: I would like see the authors’ view on whether the term ‘anaerobic threshold’ should still be used, even if that’s still common in this field? See for a review https://pubmed.ncbi.nlm.nih.gov/33112439/. The term gas exchange threshold perhaps best captures this first threshold? You also refer in the supplementary material 2 to RCP, but this was not reported in the manuscript. Was this (RCP) only determined for the purpose of determining the VE-VCO2 slope? I fully appreciate the terminology in this field is far from standard and different labs/fields use different terms, I can see the terminology was already addressed in previous reviews, but would like to bring this up again.

Authors’ response: We would like to thank the reviewer pointing out this interesting discussion and publication. Indeed, terminology in the field of cardiopulmonary exercise testing is far from standardized. We chose to adhere to the terminology used in the Perioperative cardiopulmonary exercise testing (CPET): consensus clinical guidelines on indications, organization, conduct, and physiological interpretation [1]. As the threshold is determined by gas analyses (as opposed to i.e., lactate measurement) we added the term “ventilatory”. Nevertheless, we believe that detailed discussion about the used terminology for any of the determined thresholds is outside the scope of the current study. Perhaps an international Delphi study could be done to reach consensus about the best terminology to be used in the future.

With regard to the RCP in the guideline (Supporting information S2), this threshold was indeed determined for the VE/VCO_2_-slope determination.

Figure 1. This may be an error on my end – but the resolution of the figure is not great. Please check before publication. Same in other figures.

Authors’ response:

Apologies for the inconvenience, we will ensure that high resolution figures are included in the revision.

Line 172 – how was disagreement defined? E.g. As a 5%? 10? difference between two initials assessments?

Authors’ response:

The CPET were assessed by two observers in the same session. If the two observers were unable to reach consensus on the time-point of a particular threshold, this was deemed as disagreement (so they either agreed or disagreed in a binary fashion). To be clearer in the reporting of the manuscript, we omitted the part “**In case of disagreement between observers**” and replaced this by: **“If the two observers were unable to reach consensus…** (**line 164**)”

Line 281 – Would it make sense to change from “exercise tolerance” to CRF? I would argue that CRF is then what underpins exercise tolerance – to be ability perform a task without reaching task-failure.

Authors’ response:

Indeed, “**exercise tolerance**” was changed to “**CRF**” (**line 288**)

The discussion and conclusion should better reflect some of the results reported, in my view– specifically that “within patient variation could be as much as 4.0 mL/kg/min for VO2VAT (40.8%), 5.7 for the OUES/kg (40.3%), 4.7 for VE/VCO2VAT (13.4%), and 10.4 (37.3%) for VE/VCO2-slope when using different data-averaging intervals (see Figure 2).”

I can see this is addressed in the last part of the discussion, and also mentioned in the conclusion, but I would like to ask if this can be stressed / made more clear? For example, in the abstract you state: "nor does the choice of data-averaging interval influence perioperative risk assessment", but the results show the opposite (at least at the individual level, which to me is more important than data at the group level in this instance), see lines 263 onwards.

Authors’ response:

We agree with the reviewer that we should be more nuanced in our conclusion in the abstract. Therefore, we deleted the following part of the conclusion: “**nor does the choice of data-averaging interval influence perioperative risk assessment.**” We added the following sentence (**lines 50- 51**): **“Nevertheless, caution should be taken as the chosen data-averaging interval might lead to substantial within-patient variation for individual patients.”**

**Reviewer #5:**

My perception is that the study is very well organized in writing and the statistical methods were correctly used. However kindly allow me one point to address, as per below:

The authors should also include the effect size in the statistics. This will give a more accurate picture of what the findings represents.

Authors’ response:

The effect size was embedded within the methods section (**lines 188-190**): “**Effect sizes were estimated by calculating the eta squared (i.e., sum of squares of the effect divided by the total sum of squares).”** Effect size results were added in the results section (**lines 231-232**): **“The effect sizes for all variables were ≤0.009.”**

In addition, the following was added to the discussion (**lines 306-307**): “**The observation that the differences in the mean values of the VE/VCO_2_-slope are not clinically relevant is also supported by the very small effect size (0.009).”**

Another point to emphasize is that the discussion refers to the main finding of the study, which is very appropriate. This type of initiative will always be very welcome since post-surgical complications can be significantly affected (reduced) by the use of appropriate preoperative procedures, bearing in mind that the aim of this study is to manly investigate the methods which are presently being clinically used.

Authors’ response:

We thank the reviewer for his kindness in acknowledging the relevance of the current study.

References

1.	Levett, D.Z.H., et al., *Perioperative cardiopulmonary exercise testing (CPET): consensus clinical guidelines on indications, organization, conduct, and physiological interpretation.* Br J Anaesth, 2018. **120**(3): p. 484-500.
